# CEREBRAL VISUAL IMPAIRMENT: WHEN THE EYES CAN SEE BUT THE BRAIN DOES NOT FIND

**DOI:** 10.3389/frym.2025.1561390

**Published:** 2025-09-02

**Authors:** Marinke J. Hokken, Corinna M. Bauer

**Affiliations:** 1 Department of Neuroscience, Erasmus MC, Rotterdam, Netherlands; 2 Royal Dutch Visio, Huizen, Netherlands; 3 Department of Experimental Psychology, Utrecht University, Utrecht, Netherlands; 4 The Lab of Neuroimaging and Vision Science, Department of Radiology, Massachusetts General Hospital, Harvard Medical School, Boston, MA, United States; 5 Department of Ophthalmology, Harvard Medical School, Boston, MA, United States

## Abstract

Cerebral visual impairment (CVI) is a visual disorder caused by brain damage that makes it difficult to process information from the eyes. Although their eyes may work fine, children with CVI often struggle to find and recognize objects, especially in messy or busy places. Clinicians, like eye specialists (who study eye and visual functions) and neuropsychologists (who study brain functions) work to identify children with CVI and to support them if they do. One helpful test is a visual search task, which shows how children look for things. Why is searching difficult for children with CVI? Scientific researchers use tools like eye tracking, which shows where children look during a search, and brain imaging, which helps them understand how parts of the brain work together. By combining clinical practice and scientific research, we can better understand how children with CVI experience the world and find new ways to help them in daily life.

## WHAT IS CEREBRAL VISUAL IMPAIRMENT?

Did you know that the most common visual disorder in children has little to do with damage to the eyes? It is called **cerebral visual impairment** (CVI). CVI happens when the brain struggles to understand visual information that it receives from the eyes, even though the eyes work perfectly fine. CVI can occur in children who were born very early, have genetic conditions, or have suffered a brain injury, such as a stroke or a traumatic head injury.

Your brain and eyes are a team. Your eyes help you see objects clearly, but your brain does a lot of the hard work to figure out what you are looking at. For example, your brain helps you to name an object (identification skills), remember it (memory skills), or even grab it with your hands (visuomotor skills). In children with CVI, some of these skills do not work the way they should. But CVI is different for every child because there are so many visual skills! One of the biggest challenges children with CVI face is searching for and finding things.

## HOW CHILDREN WITH CVI SEARCH FOR THINGS

Why is searching for things so hard for children with CVI? To explain, let us first think about how children *without* CVI find things. Have you ever looked for your friend in a big crowd or searched for your favorite shirt in a pile of clothes? That is called visual search, and we do it all the time without even thinking about it. When you try to find your friend in a crowded room, your eyes quickly scan the scene, sending a series of sharp, colorful images to your brain. But with so many faces around, it is too much to focus on all at once! That is where your brain steps in—it filters out the faces and details that are not important right now. This skill is called **visual selective attention**. Once the non-important information is filtered out, your brain uses your visual identification skills (which help you recognize your friend’s face) and your visual memory skills (which help you remember what your friend looks like) to find the person you are looking for. Although this may sound like a lot, most children can do this within a second! However, some children with CVI see too much information at once. This is overwhelming, like trying to find a friend in a photo where every face looks similar. Other children with CVI might see only small pieces of the scene, making it difficult to understand the whole picture. Either way, searching for things is often tiring for children with CVI, and they need more time or help to find what they are looking for.

In this article, we explain how **clinicians** and researchers work together to better understand children with CVI.

## HOW DO CLINICIANS SEARCH INVESTIGATE CVI?

**Figure 1:**
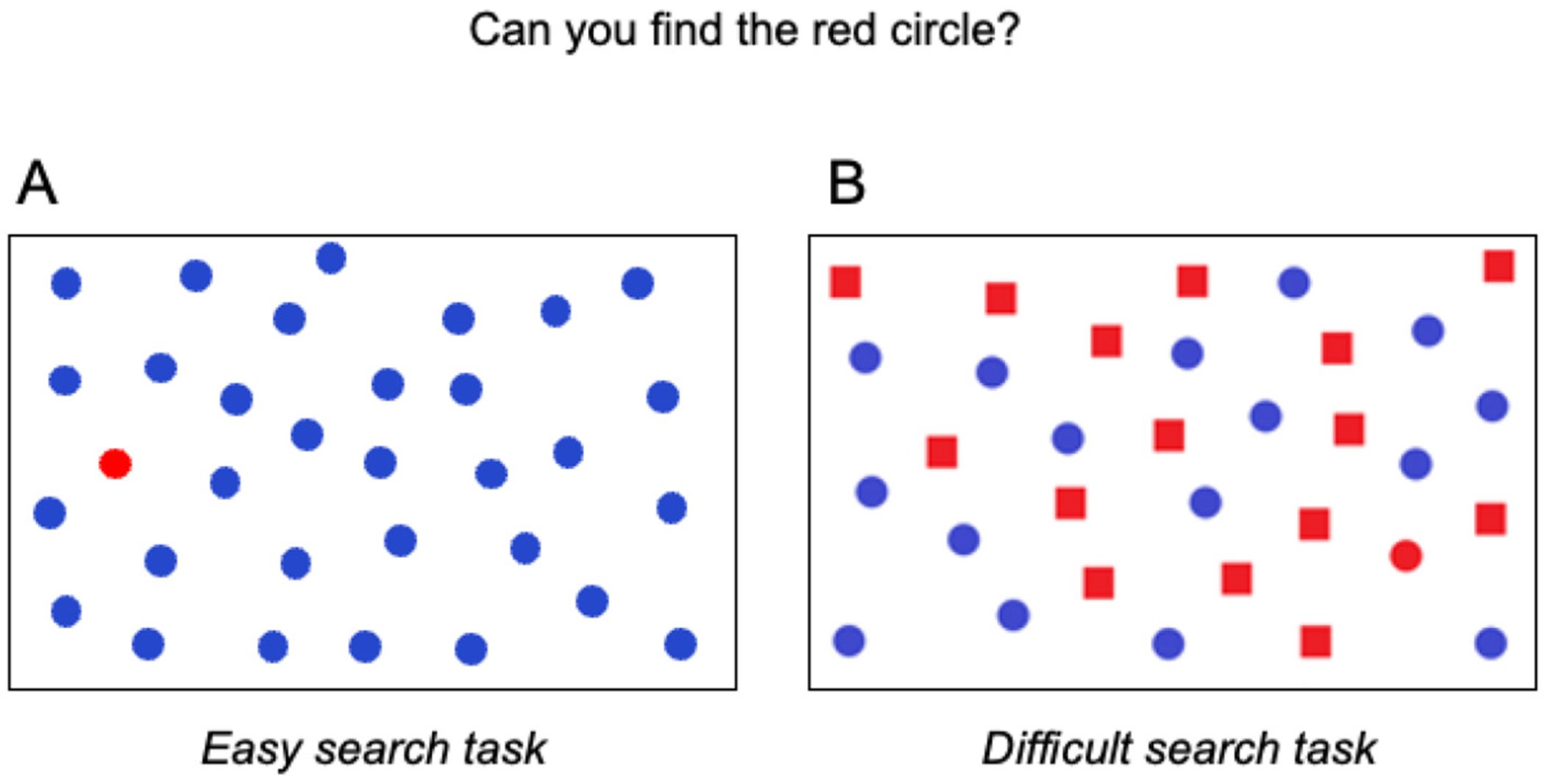
Two examples of visual search tasks. There are many different types of search tasks. **(A)** In this task, the target (red circle) is easy to find because it stands out from the distractors (blue circles). You can spot it quickly because it looks different. **(B)** In this task, the target is harder to find because the distractors (blue circles and red squares) look like the target in both color and shape. This makes it trickier to find the target, as you need to look more carefully at each shape to find the right one.

When parents or teachers think that a child might have CVI, they meet with a special team of clinicians that includes an eye specialist, who investigates eye functions, and a **neuropsychologist**, who investigates how brain damage might change the way a child learns, remembers, or sees the world. To figure out how children search for things, neuropsychologists may use **visual search tasks** (see Figure 1 for two examples). The child receives a paper filled with images of objects or shapes and is instructed to find a specific item, like a red circle. This item is called the target. The other shapes are called distractors—like the blue circles and the red squares. The child must either say as quickly as possible whether the target is present (verbal response) or must cross out the targets with a pencil (motor response). Afterwards, the neuropsychologist checks how many targets the child found and how quickly the child found them.

There are some disadvantages to visual search tasks. First, they only tell us about the result—like how fast or accurate the child is—but not *why* the child performed poorly. We do not have answers on questions like “What strategies did the child use?” or “What parts of the brain might be causing this difficulty?”. Second, because visual search tasks require a verbal or motor response, they are not suitable for very young children or children with motor or speech difficulties. Many children with CVI face these additional challenges, which makes it even harder for them to complete visual search tasks. Scientific research may help overcome these challenges in clinical practice.

## HOW DO RESEARCHERS STUDY CVI?

Researchers compare large or small groups (like twins) with and without CVI to learn more about their visual behavior. Researchers can use special technology, like **eye tracking** and brain scans, to figure out why children with CVI have trouble with visual search. This technology does not require children to speak or move.

### Eye Tracking

Eye tracking helps researchers understand where and how people look at things. An eye tracker is a special camera that follows the movement of your eyes and shows exactly where you are looking. With eye tracking, researchers can figure out where children look first, how long it takes for them to find the target, and where their eyes move after spotting the target.

**Figure 2:**
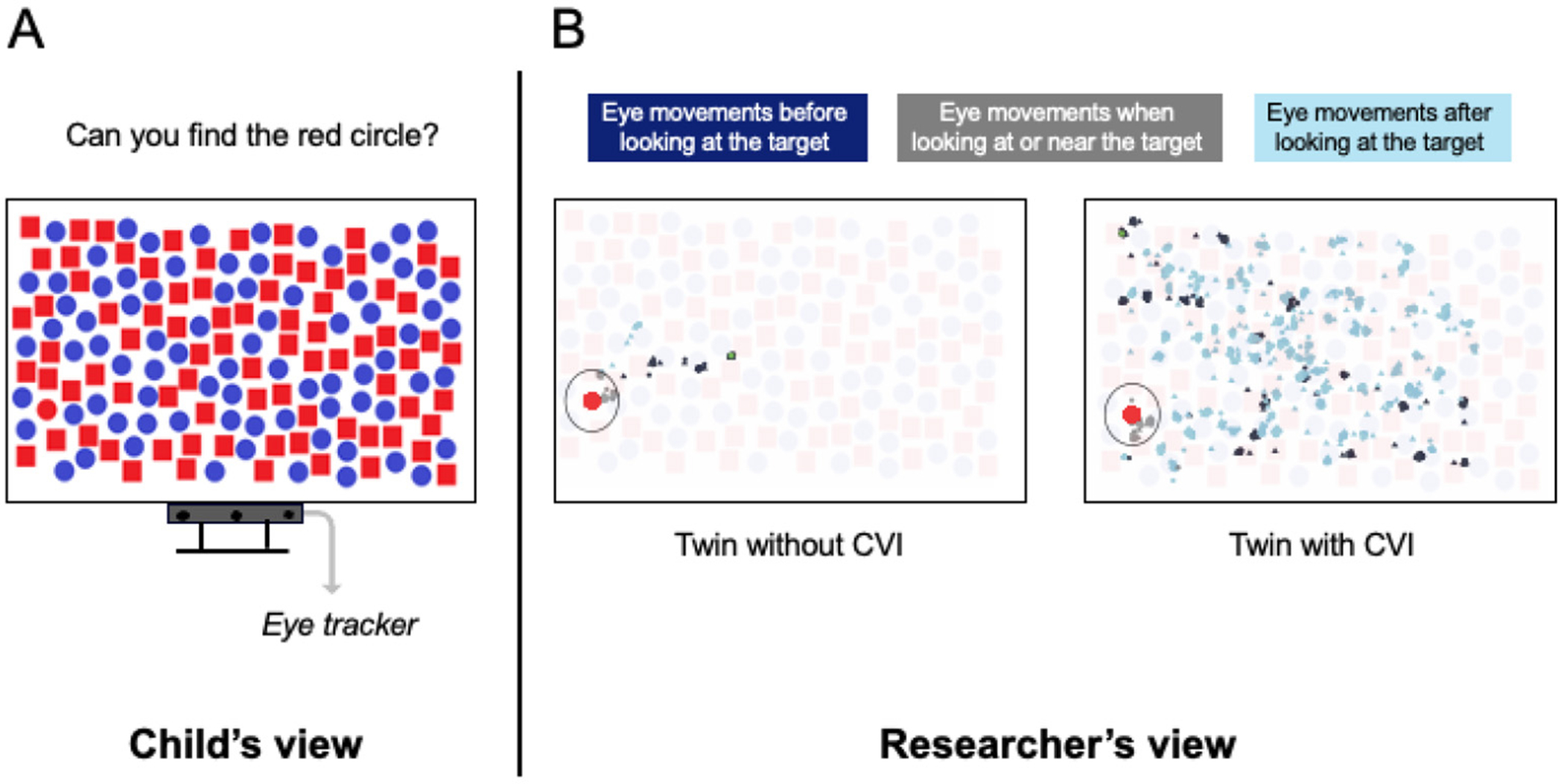
How we use eye tracking in visual search tasks. **(A)** The child sits in front of a screen with a search task on it. Below the screen is an eye tracker that follows the child’s eye movements. **(B)** The researcher, sitting nearby, watches a different screen to see exactly where the child is looking: before the child finds the target (dark blue), when the child looks at the target (gray), and after the child finds the target (light blue). This figure shows the eye movements of twin brothers aged 11 years—one with CVI and one without.

Researchers found that children with CVI need more time to find a target [1, 2]. Figure 2 shows the results of a twin study. While the twin without CVI found the target quickly, the twin with CVI looked all over the screen before finally finding the target [2]. Sometimes, children with CVI even look directly at the target but do not recognize it. That is why the twin with CVI kept searching even after looking at the target (light blue lines in the figure). This could be because their brain’s filter, the visual selective attention, is not working properly. They see so many details all at once that the target gets lost in the crowd! It could also be because their visual identification and memory skills are not working well, so when their eyes focus on the target, they are slower at recognizing it.

### Brain Imaging

**Figure 3:**
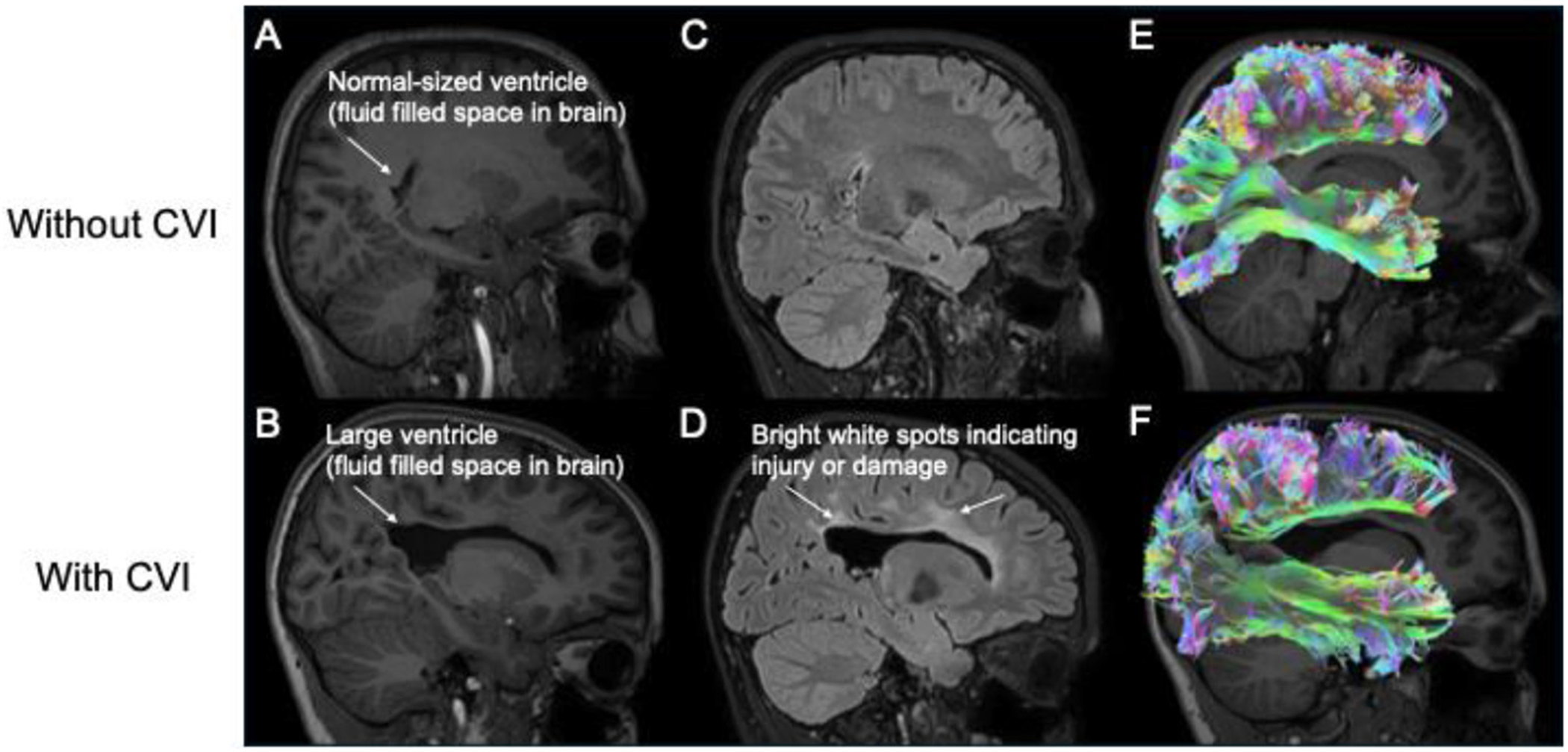
How we use MRI to look at the brain structure, areas of injury, and pathways. The eyes and nose on the right side of each image. **(A, C, E)** A 12-year-old without CVI. **(B, D, F)** A 10-year-old with CVI who was born early and had brain bleeds around the time of birth. **(A, B)** Large black space in the middle of the brain (arrow). **(C, D)** Bright white spots suggest areas of injury or damage [arrows in **(D)**]. **(E, F)** Examples of brain pathways used to process visual information. In CVI, these pathways can be damaged or smaller.

**Brain imaging**, which involves using technology to generate a picture of the brain, helps researchers to better understand how the brain works in children with CVI. Researchers use different types of brain imaging tools, such as magnetic resonance imaging (MRI) or electroencephalography (EEG), to see the structure and activity of different brain regions (Figure 3).

MRI takes detailed pictures of the brain’s structure, producing an image of what the brain looks like inside. EEG measures the electrical signals that groups of cells in your brain, called neurons use to communicate with one another. Many parts of the brain process visual information and are involved in visual attention. These specialized brain regions are far away from each other but are well connected through a series of pathways that allow them to communicate quickly and efficiently (Figures 3E, F). You can think of the pathways between brain areas like how large cities have a lot of big highways for cars to travel rapidly across large distances, and smaller streets where cars can move more slowly for shorter distances. In children, information on these brain highways typically travels quickly and efficiently. But if a brain injury damages these pathways (as indicated by the enlarged ventricles in Figure 3B, bright spots in Figure 3D, and reduced pathways in Figure 3F), it becomes difficult for the information to get through. This is what researchers think might be happening with children who have CVI: their brains have a hard time sending and receiving information about the visual world because of damage to the highways and connections [3, 4].

Brain imaging techniques called functional MRI (fMRI) and EEG are used to learn about the brain’s activity, helping researchers understand how the brain works during specific activities, such as visual search tasks. Using these techniques, researchers have discovered that the brains of children with CVI process visual information differently [1]. Their brains may become more or less active compared to typically sighted children, depending on a specific task. Researchers think this might be related to the ability of brain regions to send and receive specific information in the correct order. Sometimes having too much information all at once can make it difficult to know which is the real signal and which is not. Similarly, if the information is sent in the wrong order, it may not make sense. The same may be true in the brain following brain injury.

## WHAT WE NOW KNOW AND HOW IT CAN HELP

In sum, many children have CVI, but their visual problems differ from one child to another and can be hard to figure out. Visual selective attention difficulties are just one way that children with CVI can be impacted. Clinicians work to identify children with CVI and support them in daily life. Researchers investigate how children see their world (using eye tracking) and why they see it that way (using brain imaging). By joining forces, clinicians and researchers can better recognize children with CVI, understand their visual challenges, and learn how these affect their daily activities. This teamwork helps create improved assessments—like suitable tasks for young children—and new interventions—such as training programs and strategies to make everyday tasks easier. This way, clinicians and researchers can help children with CVI better navigate their world.

## Abbreviations

CEREBRAL VISUAL IMPAIRMENT: A type of visual impairment that is caused by brain damage or abnormal brain development, even though the eyes may work fine.
VISUAL SELECTIVE ATTENTION: A brain function that helps you focus on what you need to find and ignore unimportant things around it.
CLINICIANS: People who work in health care to help children and adults with their health, for example doctors, therapists, or psychologists.
NEUROPSYCHOLOGIST: A type of clinician who studies how the brain works and how it affects thinking, behavior, and learning.
VISUAL SEARCH TASKS: A test on paper or a screen to measure visual selective attention, where you have to search for an object or shape between other shapes.
EYE TRACKER: A special camera that follows your eye movements to see where and how you look at things.
BRAIN IMAGING: Technology that generates images of the brain’s structure to see how it looks or how regions are connected. Brain imaging can also be used to record the brain’s activity to investigate how it works.
